# Comparing the Healing Abilities of Fluorapatite and Hydroxyapatite Ceramics in Regenerating Bone Tissue: An In Vivo Study

**DOI:** 10.3390/ma16175992

**Published:** 2023-08-31

**Authors:** Leszek Borkowski, Mariusz Jojczuk, Anna Belcarz, Marta Pawlowska-Olszewska, Joanna Kruk-Bachonko, Radoslaw Radzki, Marek Bienko, Tymoteusz Slowik, Tomasz Lübek, Adam Nogalski, Grazyna Ginalska

**Affiliations:** 1 Chair and Department of Biochemistry and Biotechnology, Medical University of Lublin, Chodzki 1, 20-093 Lublin, Poland; 2 Chair and Department of Traumatology and Emergency Medicine, Medical University of Lublin, Staszica 11, 20-081 Lublin, Poland; 3 Department of Animal Physiology, Faculty of Veterinary Medicine, University of Life Sciences in Lublin, Akademicka St. 12, 20-950 Lublin, Poland; 4 2nd Departament of Radiology, University Hospital of Lublin, Staszica 16, 20-081 Lublin, Poland; 5 Experimental Medicine Center, Medical University of Lublin, Jaczewskiego 8, 20-090 Lublin, Poland

**Keywords:** fluorapatite, bone defect, bone regeneration, rabbits, implantation, imaging techniques, new bone formation, X-ray measurements

## Abstract

Some reports in the literature show the advantages of fluoride-containing apatite ceramics over hydroxyapatite (HAP), at least in some aspects. While HAP has been used extensively in the treatment of bone defects, fluoridated apatite has hardly been tested in vivo. In order to verify the biological properties of fluoride-doped apatite and to assess its therapeutic potential, we synthesized fluorapatite (FAP) and applied it as a filling in bone defects of experimental animals (rabbits). The treatment effects were evaluated on extracted bones after 3 and 6 months from implantation using peripheral quantitative computed tomography (pQCT), dual-energy X-ray absorptiometry (DXA), radiography (X-ray) and histological staining. The study proved the integration between FAP and the bone tissue, thus indicating its stimulating effect on new bone formation and mineralization. The results achieved after 3 months of treatment were difficult to interpret unequivocally and suggested the transient delay in FAP integration of bone in comparison with HAP. The reasons for this phenomenon are unclear. Most likely, these differences between FAP and HAP resulted mainly from the different porosities, densities and ionic reactivity of the ceramics, which in our opinion affected their solubility, integration and degree of bone tissue resorption. However, it was shown that 6 months after implantation, similar level of bone defect regeneration was achieved for both FAP and HAP. In this article, we present our hypothesis concerning the basis of this phenomenon.

## 1. Introduction

The process of bone replacement has been used in surgical practice for many years. Many different materials are used for this purpose, including PMMA-based bone cements. They have been extensively studied in the literature both in vitro [1,2,3] and in vivo [4,5]. Numerous apatite materials intended for the bone tissue regeneration have been tested in vivo using various animal models such as rabbits, pigs, dogs, rats, sheep, etc. [6,7]. Hydroxyapatite (HAP) and other ceramics have been repeatedly tested in animals for the treatment of bone defects [8,9,10,11,12]. Recently, attention has been paid to the use of calcium-phosphate-fluorine ceramics (fluorapatite or fluorhydroxyapatite) in medicine; however, this material has not been tested in vivo for stimulation of bone defect healing.

In the literature, there are many studies indicating that the use of fluorapatite in regenerative medicine may be beneficial. Wu et al. revealed that fluoride-doped apatite can increase collagen synthesis [13]. Fluoride-substituted apatite can improve osseointegration according to the results of an in vitro study [14]. In the study by Harrison et al. [15], it was noted that embryonic stem cells viability in the medium after 48 h of incubation with fluoridated apatite was higher than in the presence of HAP. Subsequent studies showed that fluoride ions released from bone implants can stimulate the proliferation and differentiation of osteoblasts [16,17], and accelerate the bone mineralization process [18]. In view of the above, in our opinion, fluorapatite deserves to be tested in an in vivo study to confirm (or exclude) a beneficial effect on bone tissue regeneration.

Recently, we presented the properties and biological potential of a new fluoride-substituted apatite (FAP) dedicated for application in orthopedics and bone tissue engineering [19]. In-depth study of the material including PXRD, SEM, TEM, FTIR, Raman spectroscopy and solid-state NMR analysis was published elsewhere [20]. Our studies showed that FAP calcined at 800 °C was a porous material which slowly released fluoride ions, supported osteoblast proliferation, and was non-toxic to preosteoblastic cells (viability of cells incubated with sample extracts was high). It is worth noting that the method for FAP production is novel and described in the Polish patent no. 235803.

The purposes of this study were to evaluate the capability of FAP in bone defect healing, to assess its safety, and to potentially expand the use of fluoride-containing materials in orthopedics and regenerative medicine. Therefore, we conducted an in vivo study in an animal model (male rabbits) where FAP granules were implanted into critical-size bone defects of the tibia to assess new bone formation and the degree of mineralization. Additionally, we applied HAP granules as a reference material to compare the properties and biological potential between less popular fluoride-substituted apatite and widely used non-substituted hydroxyapatite. 

## 2. Materials and Methods

### 2.1. Preparation of Materials

FAP granules were synthesized in a laboratory of the Chair and Department of Biochemistry and Biotechnology (Medical University of Lublin, Lublin, Poland), according to the procedure described in the Polish patent no. PL 235803. The following substrates have been used for the synthesis of FAP: calcium hydroxide (Ca(OH)_2_; Acros Organics, Verona, Spain), orthophosphoric acid (H_3_PO_4_; Chempur, Piekary Śląskie, Poland) and sodium fluoride (NaF; Chempur, Poland). The mixture of H_3_PO_4_ and NaF with [P]/[F] a molar ratio of 3:1 was added to a 10.8-fold amount of aqueous Ca(OH)_2_ suspension with continuous stirring at room temperature. The precipitate was allowed to age for 4 days, then washed several times with deionized water and dried at 37 °C. Finally, the FAP precipitate was calcined at 800 °C for 2 h in a muffle furnace. HAP ceramics made using the same method without the use of NaF served as a reference for the FAP material. In this study, we used apatite granules with a diameter of 0.2–0.3 mm. The process of producing FAP and HAP granules and their properties have been described in previous publications [19,20]. Basic characteristics of both materials are summarized in Table 1.

### 2.2. Experimental Design and Surgical Procedure

All research protocols were approved by the Local Ethics Committee (agreement number LKE-70/2016) and the experiment was conducted in accordance with the provisions on animal protection. The implantation procedure was planned and performed according to some current references [21,22,23].

The in vivo experiment was carried out on a group of male New Zealand white rabbits who entered the study at the age of 6 months and with an average body weight in the range of 2.5–2.8 kg. The 52 animals were placed in the animal facility of the Experimental Medicine Center of the Medical University of Lublin, were bred in optimal environmental conditions appropriate to the species (temperature, humidity, and light exposure) and fed with complete feeding and constant watering capacity. 

After a one-week adaptation period, rabbits were randomized into 3 groups:

- Control: Rabbits that have undergone a “sham surgery”, with an empty hole in the bone left to heal (n = 10);
- FAP: Rabbits that have implanted FAP granules (n = 18);
- HAP: Rabbits that have implanted HAP granules (n = 18).

Control, FAP and HAP groups were further divided into 2 subgroups according to the time of healing periods, 3 months and 6 months, resulting in the following groups shown in Table 2.

**Figure 1:**
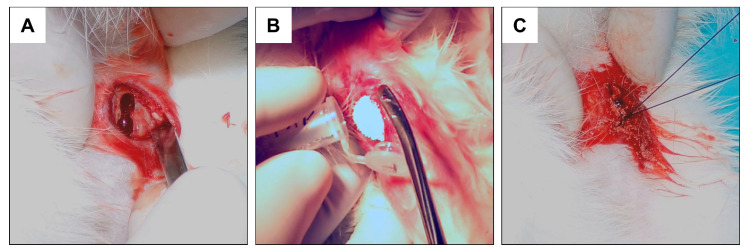
Subsequent stages of implantation of the material. (**A**) Intraoperative photograph of the rabbit tibia cavity drilled with a 4 mm diameter drill bit; (**B**) implantation of the granules into the created bone defect; (**C**) skin suturing.

Before surgery, rabbits were subjected to analgosedation via inhalation with 5% isoflurane in 95% oxygen, and then maintained under anesthesia under 3–4% isoflurane in 96–97% oxygen. Rabbits under anesthesia were transferred to the operating table equipped with a heating mat and body temperature monitor. After injecting the first group with anesthesia, animal hair was removed from the operating field using an electric razor, and then the operating field was disinfected with 96% isopropyl alcohol, which is generally used for disinfecting the operating field in humans. The skin and fascia were incised with the help of a scalpel blade no. 15 at a length of 2 cm; hemostasis was performed and the appropriate point for making the hole was located. During the procedure, 9 mm and 4.5 mm wide holes were made in all the animals using a 4.5 mm cortical drill, also used in orthopedics and human traumatology. The holes were made in the proximal end of the tibia on the medial side in the area of the so-called goose foot, the place of attachment of the knee flexors. The implant sites were chosen approximately 1.5 cm below the growth cartilage to avoid damaging it. The holes were similarly made in the area of the left lower limbs and filled with FAP or HAP granules, as shown in Figure 1. In each rabbit, 1 hole was made in 1 tibia.

After implantation of the ceramics, the fascia of the goose foot area was tightly sutured with a 3–0 absorbable thread to prevent subsequent material migration, and then the skin was sutured with a 3–0 dermal thread. The instruments used for surgical procedures were sterilized in accordance with the rules adopted in the orthopedics of the operating theater. In the Control group, the hole was left to heal itself. Immediately after the surgery, radiographs of the tibiae in the lateral (L) view were performed in all subjects in vivo. After the given period (3 or 6 months), the rabbits were slaughtered, the lower limb bones of the operated area were dissected, and the tibia bones intended for research were cleaned of residual soft tissues. Qualitative and quantitative analyses of the healing process were performed in each group on the bone samples prepared this way.

### 2.3. Qualitative Analysis of Bone Microstructure Changes

Imaging examinations are methods that allow for the observation of changes in the operated bones in vivo. X-ray is a recognized diagnostic method to assess the process of bone healing or its absence. Comparative analysis of X-ray images obtained on the first day and 3 and 6 months from the fracture provides a qualitative assessment of the healing process. The process of reducing or increasing bone density is directly reflected in the weakening of the X-ray beam, manifested as radiolucency and radioopacity, respectively [24]. Moreover, imaging studies at three and six months are the typical standards used in human bone union healing protocols. The X-ray examination after three months is the limit defined as delayed union. After six months, the bone replacement material is physiologically remodeled. We used a similar protocol in our research group [25].

#### 2.3.1. X-ray Imaging—Conventional Radiography

It is worth mentioning that the radiological pictures were taken in vivo in the lateral view of all operated bones immediately after the surgery while the anesthesia was still in effect. Based on the radiographs, the location and repeatability of the drilled bore, the degree of filling with the bone substitute material in the groups in which it was administered, and the presence of any iatrogenic refractions that may have emerged during the procedure were assessed.

Radiologic evaluation of the healing process was documented and performed posthumously in each group (Control, FAP and HAP groups: after 3 and 6 months; reference animals: after 1 day). After bone isolation, the radiographs showed all the proximal tibia ends in three projections: posterior anterior (P-A), lateral (L) and oblique (O).

The radiographs were taken with a Planmeca Intra X-ray machine ((Planmeca Oy, Helsinki, Finland)) with a high-frequency generator (66 kHz) via a Toshiba D-0711 SB lamp (Toshiba, Tokyo, Japan) (focus 0.7 × 0.7 mm). CCD sensor with dimensions of 24 × 42 × 6 mm, pixel size of 19/38 μm with a closed sensor of 940 kB was used. Anode voltage was in the range from 50 to 70 kV, anode current value was in the range from 2 to 8 mA, and exposure time was from 0.01 to 3.2 s. The electrical exposure conditions repeated for all radiographs were as follows: 60 kV, 5 mA, 0.05 s, and 0.25 mAs.

#### 2.3.2. Histology

For histological analysis, 2 cm fragments of the proximal part of the tibia were used. The bone material was decalcified in EDTA solution, embedded in a Cryomatrix gel and then frozen in liquid nitrogen. The frozen bone material was cut in cryotome into slices of 10–12 μm. The sections were stained with Harris hematoxylin and eosin (Sigma-Aldrich, St. Louis, MO, USA). Microscopic images were collected using a Olympus CKX53 light microscope (Olympus, Munster, Germany), magnification ×400, and a camera Olympus EP50 (Olympus, Munster, Germany).

### 2.4. Quantitative Analysis of Bone Microstructure Changes

Tissue bone analysis using the DXA method is a recognized procedure and considered to be the “gold standard” in densitometric measurements. This method is widely used in osteoporosis diagnostics, allowing for the determination of the current state of bone mineralization. In our studies, isolated tibia bones were evaluated. The application of this method allowed for the determination of density and mineral content of the examined bones in relation to the osteointegration of bone substitute material. Despite the fact that creating an opening and introducing the evaluated bone substitute material was limited to a specific location and its size was small, it does impact the metabolism of the entire bone. It should be emphasized that in the DXA examination, the bone is treated as a whole, and the measurements performed are two-dimensional (2D). Therefore, the authors considered it appropriate to analyze the damaged area of the bone using peripheral quantitative computed tomography. This method enables the measurement of real volumetric bone mineral density (vBMD) and mineral content (BMC) in a three-dimensional (3D) system. pQCT allowed for an understanding of the intensity of obliteration within the damaged bone area and the incorporation of the bone biomaterial material [25].

In the available literature, a period of 6 months is the most suggested as necessary for proper osteointegration. This applies to both dental implants [26] and other bone substitute materials [27]. In our studies, the bones were assessed after 3 months, which is halfway through the recommended period of proper osseointegration, and after the recommended 6-month period after the implantation of the material.

#### 2.4.1. Dual-Energy X-ray Absorptiometry (DXA)

The DXA test was performed on whole bones isolated from animals using a Norland Excell Plus densitometer (Fort Atkinson, WI, USA) combined with Norland Illuminatus Software Rev. 4.3.1 Small Subject Scan. The scanning parameters included a resolution of 3.0 × 3.0 mm with a scanning speed of 10 mm/s. Calibration of bone mineral density measurements was performed using the hydroxyapatite phantom provided by the manufacturer. The quantitative analysis of DXA was performed in each of the groups after three and six months and in the reference group by analyzing the bone mineral content, BMC (in grams), and bone mineral density, BMD (g/cm^2^). 

#### 2.4.2. Peripheral Quantitative Computed Tomography (pQCT)

The pQCT study was performed only in the part of the bone subjected to the implantation procedure using the XCT Research SA Plus apparatus (StratecMedizintechnik, GmgH, Pforzheim, Germany) combined with the Stratec v.5.50D software. Initial scanning was performed for the proximal end of the femur in 0.25 mm slices with a table travel of 7–10 mm/s (PITCH). The Region of Interest (ROI) was then selected at the site of the widest section of the bone defect where pQCT quantification was performed. The ROI fragment included the cortical and spongy bone layers and bone substitute material in the research groups. During analysis, the total bone mineral density (TOT-DEN, mg/cm^3^) and total bone mineral content (TOT-CNT, mg/cm) were assessed. 

### 2.5. Statistical Analysis

Data analysis was performed using GraphPad Prism Software (version 7.04). One-way ANOVA followed by Tukey’s multiple comparison test was applied to determine statistically significant differences between the tested groups.

## 3. Results

During the study, all 52 rabbits survived the surgery and the entire period of treatment without any complications. Based on the preliminary radiographs, a similar size of the drilled holes was found. The bone substitutes in each case were applied in a similar volume, which resulted in the complete filling of the bone defect.

### 3.1. Qualitative Analysis of Bone Substitute Materials

**Figure 2:**
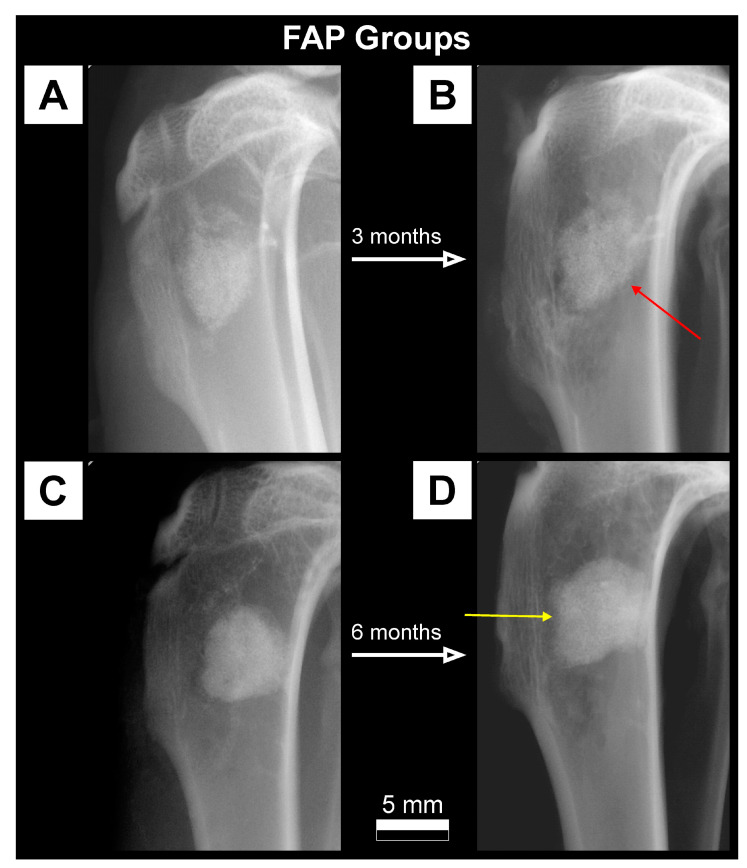
Lateral radiographs of a defect in the proximal tibial metaphysis filled with FAP granules immediately after the procedure (**A**) and after 3 months (**B**) as well as immediately after the procedure (**C**) and after 6 months (**D**). After three months of healing, (**B**) a stripe of low density shadow is visible on the edge of the filling (red arrow). After six months, the stripe of low density shadow is less visible. There are periosteal reactions (yellow arrow) on the margins of the filling and greater assimilation of the FAP material with the surrounding bone.

**Figure 3:**
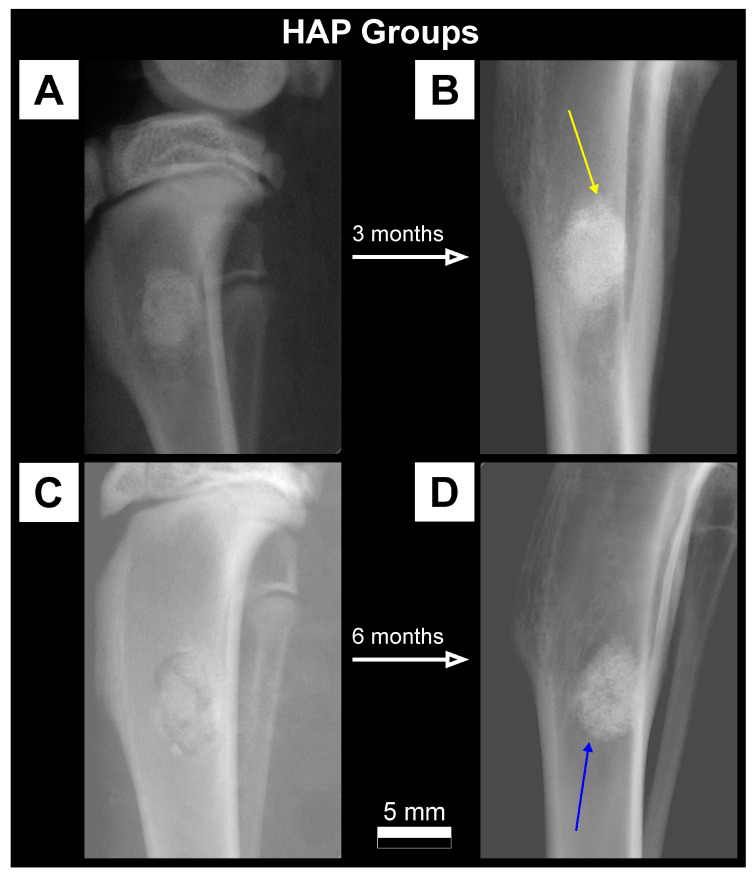
Lateral radiographs of a defect in the proximal tibial metaphysis filled with HAP granules immediately after the procedure (**A**) and after 3 months (**B**) as well as immediately after the procedure (**C**) and after 6 months (**D**). After three months, a better assimilation of the bone graft material with the surrounding tissue is visible than in the case of FAP; there are periosteal reactions on the edges of the filling (yellow arrow). After six months, the degree of radiation absorption is still higher than that of the surrounding bone tissue, but the filling area is less dense than after 3 months. Better assimilation of the defect with the surrounding bone (blue arrow).

**Figure 4:**
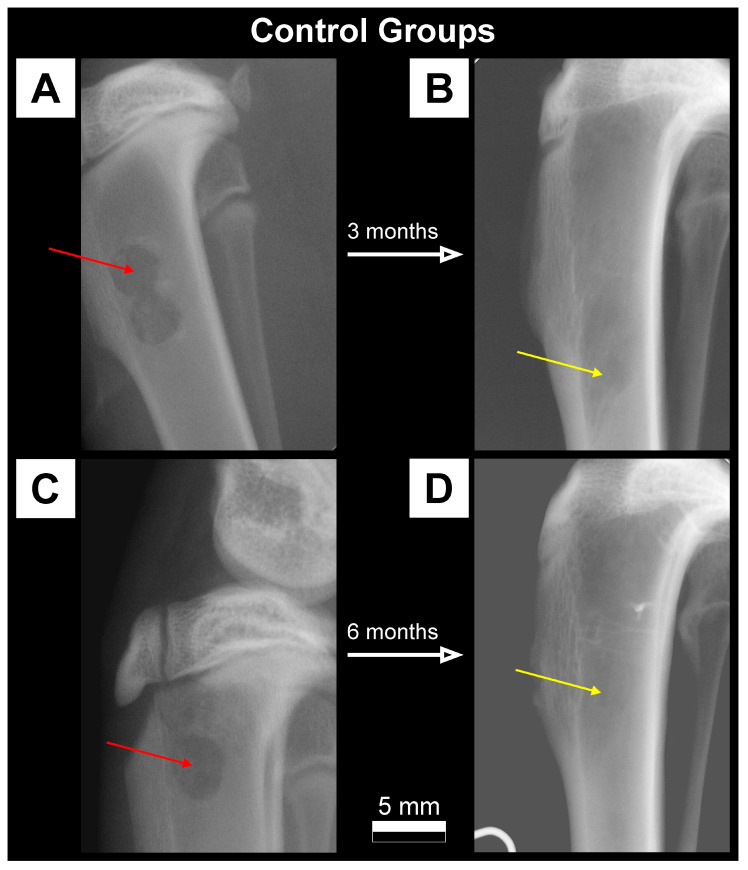
Radiographs in lateral projection of the proximal tibia metaphysis showing physiological mineralization and increase in X-ray absorption at the site of bone defect healing before/after 3 months (**A**,**B**) and before/after 6 months (**C**,**D**) in the Control group without filling the drill hole with bone substitute material. The red arrows show a radiolucency within the proximal metaphysis of the tibiae at the site of the bore. The yellow arrows show the increase in X-ray absorption at the bone defect healing site corresponding to bone formation and bone mineralization at the defect site after the healing period. There is a visible shift of the drilling site distal from the metaphysis as the rabbit grows. The degree of bone density at the defect site visibly increases and is very similar to the normal surrounding bone tissue.

Radiographic examination of rabbits tibiae during the implantation procedure showed a much higher absorption of X-rays for FAP and HAP bone substitutes than for the surrounding bone. The FAP material showed slightly higher radiopacity at the implantation site in the radiographs than the HAP material. In the Control group, without the addition of the bone material, the defect site appeared as a radiolucency area at the site of bone drilling. Lateral radiographs of rabbit tibiae from FAP, HAP and Control groups on the day of the procedure, and after 3 months and 6 months are presented in Figure 2, Figure 3 and Figure 4.

On the radiographs taken after three months in the FAP group, a stripe of radiolucency was found in all individuals in the transition zone between the bone graft material and the bone (Figure 2B). After six months, the stripe of radiolucency was less visible and there were periosteal reactions at the edges of the filling (Figure 2D). In the HAP group, periosteal reactions were observed on the border of the bone graft material and normal bone in the third month of observation (Figure 3B). In the HAP 6M group, the bone substitute material showed a slightly lower degree of radiopacity in the radiographs compared to the radiographs in the HAP 3M group (Figure 3D). In the Control 3M group, there was a normal increase in X-ray absorption at the site of the previous bone radiolucency in the area of the drill hole. There was still a difference in the degree of X-ray absorption compared to the surrounding bone (Figure 4B). After six months, the degree of radiopacity on the radiographs increased, which after this time was comparable to the surrounding bone (Figure 4D).

**Figure 5:**
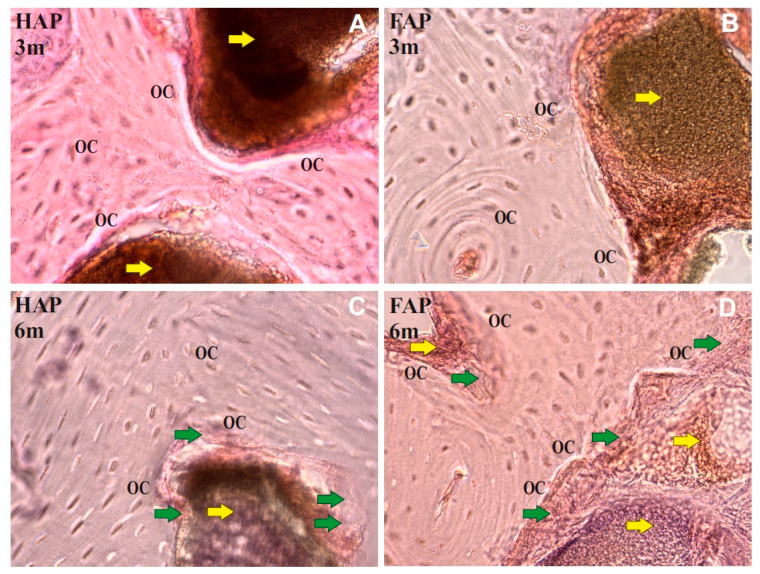
Cross-section of the shaft in the proximal part of epiphysis of the rabbits tibiae after surgery FAP or HAP granules implantation (after 3 months: (**A**,**B**), and after 6 months: (**C**,**D**)). The symbol OC marks the osteocyte, yellow arrows mark the implant, and green arrows mark the new bone.

Histological examinations showed that 3 months after the implantation of the FAP and HAP in the part of the proximal tibial metaphysis, the implanted material showed only a slight integration with the bone tissue, and no signs of inflammation were found in the tested preparations. Both in the HAP and FAP groups, it was shown that the material adhered closely to the bone tissue, and numerous osteocytes were present on the edges of the compact and spongy bone (Figure 5A,B).

Six months after implantation in the HAP group, further progressive osseointegration with the HAP in the form of a delicate plexus bone layer was found (Figure 5C). In the FAP group, more intensive osseointegration and a thicker layer of woven tissue between the FAP biomaterial and bone tissue were demonstrated in comparison to the bones filled with HAP. In the FAP 6M group, penetration of the plexus tissue into the biomaterial was found, which may indicate increased osseointegration of the biomaterial (Figure 5D).

### 3.2. Quantitative Analysis of Bone Substitute Materials

Subsequently, changes in the microstructure of the bone defect were quantified by measuring bone density and mineral content using peripheral quantitative computed tomography (pQCT) and dual-energy X-ray absorptiometry (DXA). In addition, pQCT bone cross-sections are shown in Supplementary Materials (Figure S1).

**Figure 6:**
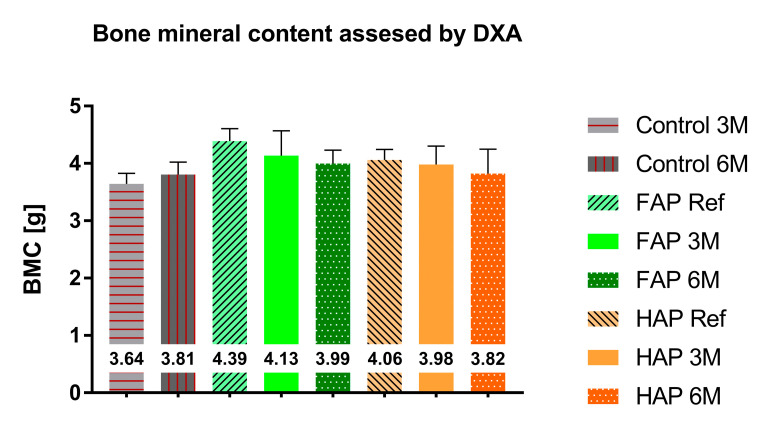
Total content of bone mineral measured via DXA. Mean values are entered in the chart.

**Figure 7:**
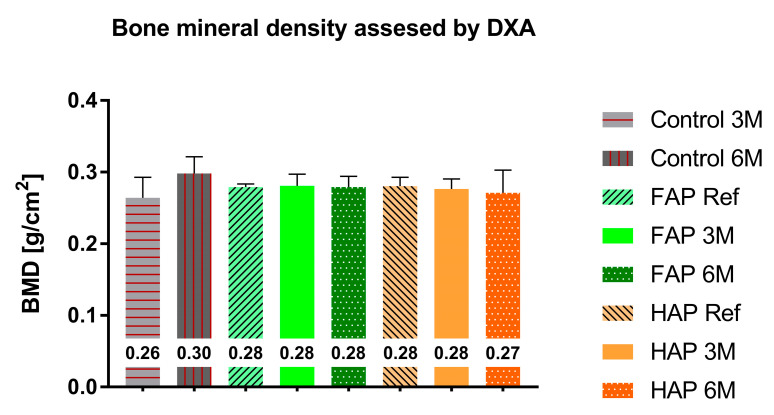
Bone mineral density measured via DXA. Mean values are entered in the chart.

The mineral content and density of whole bones obtained via DXA showed no statistically significant differences between the study groups. Tibial bone preparations filled with FAP showed a higher mineral content (BMC) compared to the group with HAP granules, 4.388 g and 4.06 g (on the 1st day of the study); 4.134 g and 3.981 g (after three months); and 3.996 g and 3.823 g (after 6 months), respectively; however, these differences were not statistically significant (Figure 6). BMD in both FAP and HAP groups was at the same level during the observation period (Figure 7). In the Control group, an increase in BMC and BMD could be observed during the experiment.

**Figure 8:**
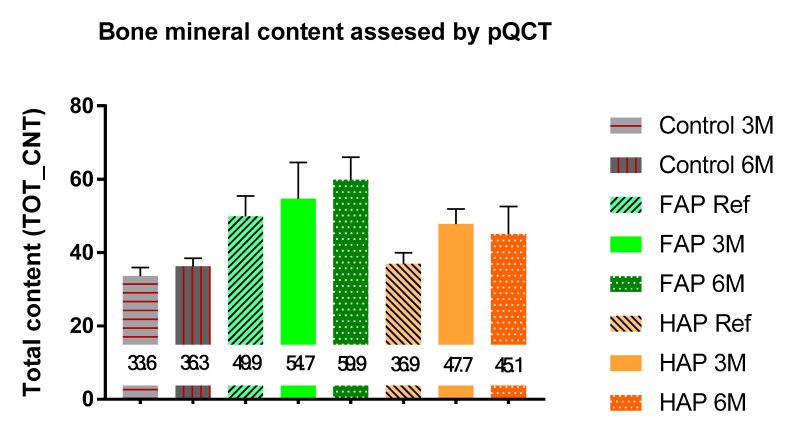
Total content of bone mineral (TOT-CNT) measured via pQCT. Mean values are entered in the chart. See the statistically significant differences of these results in Supplementary Materials, Table S1.

Quantitative analysis via pQCT showed statistically significant measurements. In the first day after implantation of ceramic granules, the mean TOT-CNT value for FAP was significantly higher (49.89 mg) than for the HAP group (36.96 mg). After 3 months, TOT-CNT increased in both groups, with a greater increase (in comparison to reference) observed in the HAP group. As a result, the difference in mineral content was not statistically significant between the FAP 3M and HAP 3M groups, while there were statistically significant differences between FAP 3M and Control 3M and between HAP 3M and Control 3M. In sixth month of the experiment, the mineral content in the FAP group increased to 59.9, while in the HAP group, it decreased to 45.1 (Figure 8 and Table S1). The mean TOT-CNT value in the Control group after three months was 33.6 mg, and after six months, it increased to 36.33 mg.

**Figure 9:**
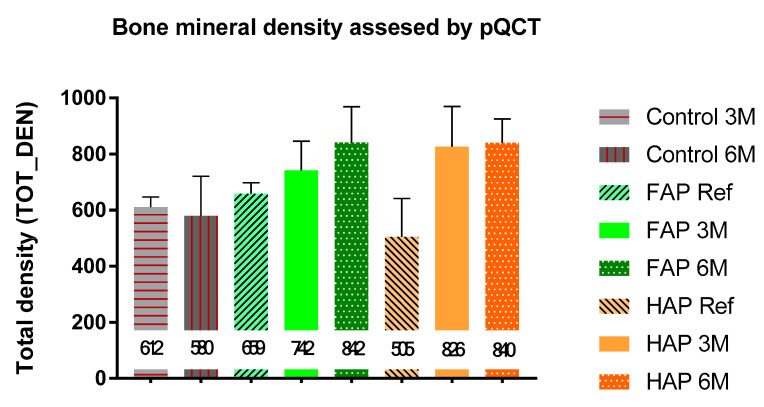
Total density of bone mineral (TOT_DEN) measured via pQCT. Mean values are entered in the chart. See the statistically significant differences of these results in Supplementary Materials, Table S2.

In the reference study, after the first day of implantation, tibial preparations supplemented with the FAP material showed a higher mineral density per bone surface (TOT-DEN) compared to the group with HAP granules, 658.8 mg/mm^3^ and 505.4 mg/mm^3^, respectively. The TOT-DEN values for both materials in the sixth month of the study were similar and amounted to 842.1 mg/mm^3^ for FAP and 840.1 mg/mm^3^ for HAP. These values were significantly higher than mineral density in the Control group, which was 579.9 mg/mm^3^ (Figure 9 and Table S2).

## 4. Discussion

Both the bone graft materials (FAP and HAP) appeared on the radiographs as areas of radiopaque with a much higher degree of radiation absorption than the surrounding bone tissue. The qualitative assessment of the radiographs showed a difference in radiopaque between FAP and HAP, with a higher degree of radiation absorption in the FAP group which results from the higher density of the FAP granules, as presented in Table 1 (Section 2). After six months of healing, in the FAP and HAP groups, there was a complete assimilation of the osteogenic material with the surrounding bone and an increase in the trabecularization of the bone structure as an expression of the parallel process of osteogenesis at the site of the bone defect. Control bones after 3 months proves the ongoing incomplete mineralization process, i.e., the formation of bone trabeculae at the site of the defect. After six months, in the area of the drill hole, the bone defect with trabecular bone healed almost completely.

In general, the FAP material integrated well with the bone tissue. After 3 months, an interesting phenomenon was observed at the bone-implant interface (Figure 2B). Stripes of low density shadow visible on the edge of the implant is the potential effect of fluoride on bone tissue. One possible hypothesis is that fluoride may cause transient osteolysis. This type of adverse reaction has been described by Pelto-Vasenius et al. and Bostman et al. for self-absorbable materials [28,29]. However, in our studies, histological analyses did not confirm the occurrence of osteolysis at or near the bone-implant interface. Another explanation for this phenomenon observed in the FAP 3M group could be formation of a zone of increased bone density in the filling area, and formatting radiolucency stripe at the transition zone. This would be in line with the reports such as Shah et al. stating that fluoride ions stimulate the mineralization of bone tissue [18]. Periosteal reactions at the edges of the implant in the FAP 6M group demonstrate a very close integration of the implant with the bone tissue, and may indicate an even greater assimilation of the FAP material with the surrounding bone than HAP. The periosteal reactions visible in the pictures reflect the healing processes of the bone tissue. The vast majority of bone tissue is located under the periosteum, which is why most of the repair cells come from the periosteum [30]. The reactions visible on the preparations correspond to the non-aggressive reactions of the occlusion stimulated by the fracture healing process [31].

Some periosteal reactions observed on the border between the bone graft material and the normal bone in the HAP 3M group indicate good integration of HAP with tissue in vivo. This observation is consistent with other studies indicating good bioactivity and high biocompatibility of HAP [32,33,34]. In the HAP 6M group, a slightly lower degree of saturation was observed. This can naturally be attributed to the process of hydroxyapatite resorption presented, e.g., by Padilla et al. [35]. Interestingly, this phenomenon was not observed after FAP implantation, which indicate less or negligible resorption of the fluoride-substituted ceramics. This may be related to lower porosity (Table 1), lower ionic reactivity and lower solubility of FAP in relation to HAP [36]. In addition, this process can also be associated with the acidification of the extracellular microenvironment that occurs not only in the case of inflammation, but also during the process of resorption of the bone tissue (or implant) with the participation of osteoclast proton pumps [37,38,39]. Simmer et al. described in detail the solubility of FAP and HAP in a reduced pH environment and demonstrated that FAP better resists acid attack [40]. 

According to DXA, the biggest difference in BMC was between the FAP Ref and HAP Ref groups. Considering the same size of bone defects in both groups and the same volume of implanted material, this difference is probably due to the density of these granules, as shown in Table 1. There was also a decrease in BMC after 3 and 6 months in both groups, which confirms the assumption about the ongoing process of apatite dissolution and resorption of implants. Observed increase in BMC and BMD in the Control group probably reflects the natural healing process of the bone defect and correlates with the X-ray images (Figure 4). The fact that DXA showed no statistically significant differences between the study groups may be due to the lower efficiency of the DXA method for small bone areas. The BMC and BMD values obtained via the DXA method is a summation measurement calculated from two-dimensional data, with overlapping anatomical structures, and the final value is averaged. A significant advantage of pQCT over DXA is the measurement of the degree of X-ray absorption for each three-dimensional element of the examined bone. This method of data analysis optimally describes the examined bone structure, as opposed to the DXA data analysis based on a set of average values of parameters characterizing the entire bone structure. The pQCT method is by far the most accurate method for measuring bone mineral density [41].

Contrary to DXA, pQCT results showed statistically significant differences between some tested groups. BMC in the FAP group continued to increase over time, while in the HAP group, it reached its highest level after 3 months, and after 6 months, there was a slight decrease. A continuous increase in BMC in the FAP group may indicate a natural increase in bone mineralization during the repair processes in the absence of significant resorption of bone substitute material. The decrease in BMC in the HAP group in the sixth month of observation, after its initial increase in the third month of measurement, most likely indicates partial resorption of the bone graft material (Figure 8). Our quantitative data obtained in CT are consistent with our qualitative evaluation of the radiographic images. In classic radiographs, the denser the bone, the greater the opacity on the X-ray image. Density assessment is therefore based on a qualitative (visual and subjective) assessment of the material. In our material, HAP 6M showed the lowest density, expressed as the greatest X-ray lucenecy, compared to HAP 3M and FAP 3M. Quantitative CT analysis (numerical) with objective assessment of bone mineral content confirmed the data obtained in the qualitative assessment (Figure 2, Figure 3 and Figure S1). Goto et al. also noted similar results on the resorption of synthetic porous hydroxyapatite and replacement by the newly formed bone [42]. Similarly, Okuda et al., who used calcium-deficient hydroxyapatite in their studies, reported the slow resorption with replacement by bone [43]. The bone mineral content (BMC) at the defect site in the Control group increased with the healing time, which indicates an advantage of bone formation at the defect site. Taking into account the increase in BMD in this group, we can assume that the natural healing process of the bone defect was dominated by bone formation with increasing bone mineral density. In the FAP and HAP groups, there was a difference in the BMD growth curve. For the HAP group, this value was close to the final value after three months, while for the FAP group, the increase was linear. These data are consistent with the qualitative analysis of the radiographs and may indicate faster primary assimilation of bone-forming material in the HAP group than in the FAP group. However, it is likely that the beneficial effect of fluorine in this case, suggested by other authors and our previous in vitro studies, could be suppressed by the higher density of FAP and lower ionic reactivity, which limited the process of bioresorption of this ceramic and resulted in the lower rate of delivery of calcium and phosphate ions to the surrounding environment. 

In further scientific work, we plan to focus on developing and testing the biological potential of composite materials containing FAP. Numerous reports indicate the advantages of adding a polymer components to various granules, powders or nanomaterials [44,45,46,47]. Composites with FAP potentially combine the advantages of apatite ceramics and polymer matrix [48,49]. Organic–inorganic composites are designed to imitate the real nature of bone, i.e., combine the ductility and plasticity of the polymer phase with the strength and biocompatibility of the inorganic phase, so as to obtain bioactive materials with improved mechanical properties and an appropriate degree of resorption.

## 5. Conclusions

The purpose of this paper was to verify the advantage of FAP over HAP in bone tissue regeneration in vivo, based on the literature and our previous in vitro studies. However, the results obtained in the animal study did not conclusively support this hypothesis, as similar bone regeneration effects were obtained for both tested ceramic materials after 6 months of treatment. These observations can be a starting point for further works related to the optimization of FAP properties or the development of a new composite material containing FAP in order to stimulate the bone tissue regeneration process.

## 6. Patents

Borkowski: L.; Belcarz, A.; Przekora, A.; Ginalska, G. Production Method for Biocompatible Implant Material. Polish Patent no. 235803, 6 October 2020.

## Figures and Tables

**Table 1:** The chemical composition and properties of FAP and HAP ceramics. Parameters were expressed as mean value (± standard deviation). Data from [19].

Parameter [Units]	Type of Ceramics		Statistically Significant Difference *
	FAP	HAP	
Fluorine content [atomic %]	4.12 (0.3)	0	yes
Solid phase density, SPD [g/cm^3^]	3.02 (0.03)	2.91 (0.02)	yes
Bulk density, BD [g/cm^3^]	0.75 (0.01)	0.55 (0.01)	yes
Porosity, P [%]	75 (0.19)	81 (0.15)	yes
Total intrusion volume, V [cm^3^/g]	1.01 (0.02)	1.47 (0.02)	yes

* Details of the statistical analysis can be found in the aforementioned publication [19].

**Table 2:** Designations of animals taking into account the treatment period with the number of animals given.

Group of Animals	Healing Period	
	3 Months	6 Months
Control	**Control 3M**	**Control 6M**
	(n = 5)	(n = 5)
FAP *	**FAP 3M**	**FAP 6M**
	(n = 9)	(n = 9)
HAP *	**HAP 3M**	**HAP 6M**
	(n = 9)	(n = 9)

* The groups were increased after including the reference animals: **FAP REF** (n = 3) and **HAP REF** (n = 3), used to determine the BMC and BMD in densitometric and tomographic measurements immediately after implantation of materials.

## Data Availability

Not applicable.

## Supplementary Materials

The following supporting information can be downloaded at: https://www.mdpi.com/article/10.3390/ma16175992/s1, Figure S1: pQCT images of bone with filling: (A) FAP 3M; (B) FAP 6M; (C) HAP 3M; (D) HAP 6M. In FAP preparations, there is an increase in the density of the bone defect at the site of filling with the duration of observation (panels A and B, red arrows). The cortical layer of the defect is almost developed (panel B - blue arrow). In the HAP preparations, the density of the bone defect filling is slightly lower in the 6th month of observation than in the 3rd month of observation (panels C and D, yellow arrows). There are slight porosities within the restoration (panel D - yellow asterisk), with a fully developed cortical layer of the defect; Table S1: Results of Tukey’s post hoc test for TOT_CNT results obtained by pQCT. Due to the large number of compared groups, only those for which a statistically significant difference was found are presented. Symbol meaning: * *p* ≤ 0.05, ** *p* ≤ 0.01, *** *p* ≤ 0.001, **** *p* ≤ 0.0001; Table S2: Results of Tukey’s post hoc test for TOT_DEN results obtained by pQCT. Due to the large number of compared groups, only those for which a statistically significant difference was found are presented. Symbol meaning: * *p* ≤ 0.05, ** *p* ≤ 0.01.
