# Supplementary material for: Comparing the Healing Abilities of Fluorapatite and Hydroxyapatite Ceramics in Regenerating Bone Tissue: An In Vivo Study
Source: Materials (Basel). 2023 Aug 31;16(17):5992. doi: 10.3390/ma16175992 (PMC10488477; doi:10.3390/ma16175992)
Supplement: Supplementary file 1 [file materials-16-05992-s001.zip › materials-2561222-supplementary.pdf]

materials

MDPI

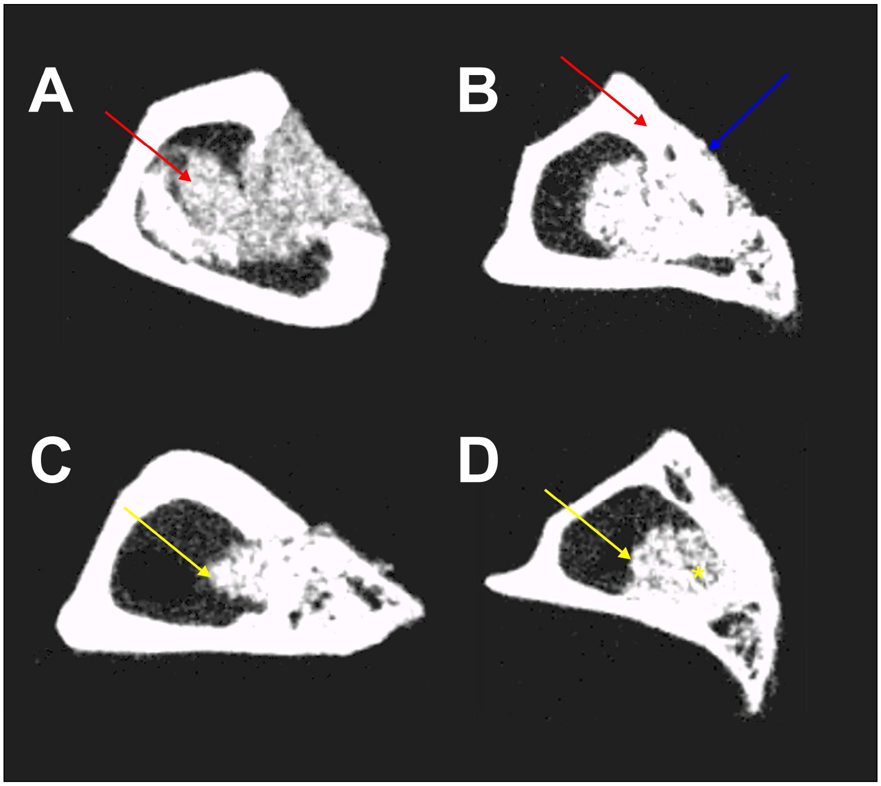

Figure S1. pQCT images of bone with filling: (**A**) FAP 3M; (**B**) FAP 6M; (**C**) HAP 3M; (**D**) HAP 6M. In FAP preparations, there is an increase in the density of the bone defect at the site of filling with the duration of observation (panels A and B, red arrows). The cortical layer of the defect is almost developed (panel B—blue arrow). In the HAP preparations, the density of the bone defect filling is slightly lower in the 6th month of observation than in the 3rd month of observation (panels C and D, yellow arrows). There are slight porosities within the restoration (panel D—yellow asterisk), with a fully developed cortical layer of the defect.

Table S1. Results of Tukey's post hoc test for TOT_CNT results obtained by pQCT. Due to the large number of compared groups, only those for which a statistically significant difference was found are presented. Symbol meaning: * $p \leq 0.05$, ** $p \leq 0.01$, *** $p \leq 0.001$, **** $p \leq 0.0001$.

Groups Compared	Significant?	Summary	Adjusted *p* Value
Control 3M vs. FAP Ref	Yes	*	0.0243
Control 3M vs. HAP 6M	Yes	*	0.0479
Control 3M vs. HAP 3M	Yes	**	0.0064
Control 6M vs. FAP 3M	Yes	***	0.0001
Control 3M vs. FAP 3M	Yes	****	<0.0001
Control 3M vs. FAP 6M	Yes	****	<0.0001
Control 6M vs. FAP 6M	Yes	****	<0.0001
FAP 3M vs. HAP 6M	Yes	*	0.0432
FAP 3M vs. HAP Ref	Yes	**	0.0031
FAP 6M vs. HAP 3M	Yes	**	0.0039
FAP 6M vs. HAP 6M	Yes	***	0.0002
FAP 6M vs. HAP Ref	Yes	****	<0.0001

Table S2. Results of Tukey's post hoc test for TOT_DEN results obtained by pQCT. Due to the large number of compared groups, only those for which a statistically significant difference was found are presented. Symbol meaning: * $p \leq 0.05$, ** $p \leq 0.01$.

Groups Compared	Significant?	Summary	Adjusted *p* Value
Control 3M vs. FAP 6M	Yes	*	0.0119
Control 3M vs. HAP 3M	Yes	*	0.0281
Control 3M vs. HAP 6M	Yes	*	0.0157
Control 6M vs. FAP 6M	Yes	**	0.0026
Control 6M vs. HAP 3M	Yes	**	0.0069
Control 6M vs. HAP 6M	Yes	**	0.0037
FAP 6M vs. HAP Ref	Yes	**	0.0011
HAP Ref vs. HAP 3M	Yes	**	0.0024
HAP Ref vs. HAP 6M	Yes	**	0.0014
